# Using theory in health professions education research: a guide for early career researchers

**DOI:** 10.1186/s12909-022-03660-9

**Published:** 2022-08-04

**Authors:** Koshila Kumar, Chris Roberts, Gabrielle M. Finn, Yu-Che Chang

**Affiliations:** 1 Prideaux Discipline of Clinical Education, College of Medicine and Public Health, Flinders University, Adelaide, South Australia Australia; 2 Education Office, Sydney Medical School, Faculty of Medicine and Health, The University of Sydney, Sydney, NSW Australia; 3 Division of Medical Education, School of Medical Sciences, Faculty of Biology, Medicine and Health, The University of Manchester, Manchester, M13 9NT UK; 4 Department of Emergency Medicine, Chang Gung Memorial Hospital; Chang Gung Medical Education Research Centre (CG-MERC) and School of Medicine, Chang Gung University, Taoyuan City, Taiwan

Theory provides complex and comprehensive explanations of a wide range of phenomena (i.e., things that we research), and using theory can enhance quality in health professions education (HPE) research [1–4]. However, those who are new to HPE research and early career researchers (ECRs) can find it challenging to use theory. In this paper, we outline key considerations (see Table 1) for using theory in HPE research, both in relation to theory as a subject or content area and the process of using it, including critically questioning which theories are priviledged in the HPE literature [5, 6]. By providing this guidance, we hope to support new and early-career HPE researchers around the globe to enhance their capacity to appraise and improve theoretical quality, both in relation to their own work and the HPE literature. While theory is the focus of this paper, we acknowledge it is one of many aspects that researchers have to concurrently balance and integrate into their work [7].

**Table 1:** Considerations for using theory in health professions education research

***1. Consider theory comprehensively and critically***
i. Clarify interpretations and terminology associated with theory including what theory means to you
ii. Seek suggestions about theory from research supervisors/mentors, experienced research colleagues, your professional networks, and/or social media
iii. Engage with theories from other disciplines (e.g., social psychology, sociology, education, philosophy, organisational, and economics etc.), cultural settings, and those that are less well established in the HPE literature, and with original sources of theory
iv. Collate, deconstruct, and discuss examples of theory use, including how other researchers situated in the same paradigm or using a similar methodology use theory
v. Question theory—where is a theory from and what are its philosophical and cultural foundations, how has it developed and evolved, who is using it and how, and what are its strengths and limitations?
vi. Keep current with new developments, debates, and discussions regarding theory
***2. Consider the alignment between paradigm and theory***
i. Reflect on and articulate your paradigm (philosophical stance) as a researcher, and how it interacts with theory
ii. Consider the philosophical foundations of your chosen theory (or multiple theories)
***3. Consider the interplay between methodology and theory***
i. Consider the philosophical foundations of your chosen methodology and how it interacts with theory
ii. Reflect on if you intend to build new theory or extend existing theory
***4. Consider the fit between theory and unit of analysis***
i. Identify the level (micro, meso, macro) at which you will examine the research phenomena of interest
ii. Clarify if your research is seeking to apply a critical or emancipatory theoretical lens in terms of examining the impact of power, race, gender, politics, history, and culture
***5. Consider when theory is used in the research process and associated implications***
i. Identify whether theory is to be used deductively (from the outset) or inductively (at the analysis stage)
ii. If deductively, outline how theoretical constructs inform the research aims and/or question/s, data collection, and analysis
iii. If inductively, outline how theory has informed the analysis
***6. Consider the complexities of how theory is written up***
i. Identify if any adaptations were made during the use of theory
ii. Identify how a theory has been extended or challenged through the research
iii. If a new theory has been generated, consider how it can be written up in a way that links to existing theory
iv. Acknowledge the complexities of balancing theory-in-use and reconstructed theory
v. Consider a fit-for-purpose placement of theory in writing up research

## Consider theory comprehensively and critically

There are many different definitions of theory articulated in the HPE literature. These include theory as: an organised, coherent, and systematic articulation of a set of issues that are communicated as a meaningful whole [1]; a means of better understanding the mechanics of the research phenomena [3], a system of ideas intended to explain a phenomenon [8]; or a ‘philosophical stance informing the methodology’ [9]. These multiple definitions highlight that there are different interpretations about what theory is. For example, some regard theory as the underpinning philosophy or paradigm of a discipline which are the assumptions which underpin what a researcher does and why (we cover this in more detail later). Others, including ourselves, regard theory as a specific set of ideas or a lens that can be applied to examining and explaining phenomena. Furthermore, there are many different terms associated with theory [2] and interchangeable use of terminology. Therefore, we advise those new to HPE research to take time in the early stages of their research to clarify interpretations and terminology associated with theory and their own understandings of theory, through discussion with their supervisors/mentors, and research colleagues.

Given the range of theories available for use, we suggest ECRs seek guidance from supervisors/mentors and colleagues about what theories they use and see which theories are being discussed in their professional networks and social media. Once there is an initial level of familiarity, we advise ECRs to note which theories are being used in the primary HPE research literature (peer-reviewed journal articles) and grey literature (e.g., reports, conference presentations etc.) in the HPE field. To reduce the reliance on others’ interpretations of theory, it will also be important to engage with original sources of a chosen theory (or theories). To enhance inclusivity and diversity of theory [5, 6], we advise ECRs to engage with theories from other disciplines (e.g., social psychology, sociology, education, philosophy, organisational, and economics etc.), cultures and geographical settings, and with theories that are less well-known in the HPE field. We also recommend ECRs collate, deconstruct, and discuss peer-reviewed examples of theory use. To demonstrate how to deconstruct theory use, we have taken a small sample of papers published in this journal and identified how theory is used in relation to paradigm, methodology, unit of analysis, timing of theory use, and write up of theory (See Table 2). Collectively, these strategies will help ECRs to identify and choose a theory (or multiple theories) that is fit for purpose.

**Table 2:** Examples of papers using theory from BMC Medical Education

Paper	Theory	Paradigm	Methodology	Unit of analysis	Timing of theory use	Write up of theory
Horsburgh, Ippolito [10]	Bandura’s theory of social learning	Interpretive	Qualitative methodology; interviews	Micro level (individual)—how learners and teachers perceive and make sense of learning from role modelling in clinical settings	Theory used to inform data analysis	Theory outlined in the Background, Methods, and Discussion
Held et al. [11]	Social network theory	Not explicity stated, but research approach is consistent with the positivist paradigm	Quantitative metodology; survey	Micro level (group processes)—student learning networks in a rural community based placement	Theory used to inform instrument development and data analysis	Theory outlined in the Background, Methods, and Discussion
O’Keefe et al. [12]	Activity theory	Not explicitly stated, but the research approach is consistent with the constructivist paradigm	Action research; interviews, focus groups and direct observation	Meso level -management of student clinical placements across three health service sites	Theory used in the data analysis	Theory outlined in the Background, Methods (specifically data analysis), and in the Discussion
Ji et al. [13]	Social netwrok theory; complex systems theory	Not explicity declared but consistent with the positivist paradigm	Quantitative metodology; analysis of publications data	Macro level—research topics and trends in medical education	Theory used in the data analysis	Theory outlined in the Background, Methods (specifically data analysis), and in the Discussion

As ECRs engage with theory, they will need to adopt a critical stance and ask questions both about theory as a subject/content area and the process of theory use. Questions that can be asked about theory as a subject/content area include: what are the origins of a particular theory; how has that theory evolved over time; and who is using that theory and how [14]? It is important to question theory because each theory: privileges a certain way of framing a research problem; is underpinned by certain assumptions; has different strengths and limitations [8]; and offers a different level of explanation and perspective [2–4, 8]. Adopting a critical stance in relation to theory is also vital to decolonise and diversify research practices [5, 6]. In order to effectively critique theory, we encourage ECRs to keep current with contemporary debates and discussions about theory and its use. So our first set of considerations (outlined in Table 1) relates to engaging comprehensively and critically with theory as a subject or content area.

## Consider the alignment between paradigm and theory

A paradigm is a world view or a ‘philosophical way of thinking’ [15] which guides what a researcher does and why. It encompasses: values and assumptions about the nature of reality (ontology); how we come to know (epistemology); the research processes (methodology); and values (axiology) [16, 17]. There is broad agreement that there are four main paradigms: positivism; post-positivism; constructivism/interpretivism; and critical theory [16–19]. Qualitative research is aligned with the post-positivist, constructivist/interpretivist, and critical paradigms, quantitative research corresponds to the positivst paradigm, and mixed methods research can involve a combination of all paradigms. A key element of HPE research relates to reflecting on and articulating one’s paradigm [17], ideally, at the time of designing a study.

Critically, ECRs are advised that although not every published study will explicitly name a paradigm, each study is situated within a specific philosophical milieu [20]. Furthermore, ECRs also need to understand that each theory itself is also underpinned by certain assumptions based not only on its disciplinary roots, but also its cultural roots [5, 6]. For example, theories derived from psychology will differ from sociological theories in terms of their fundamental philosophical assumptions about phenomena and focus on understanding individuals and/or groups (psychological theories) and social groups, communities, and cultures (sociological theories). Similarly, theories from Western cultures may priviledge certain kinds of knowledge and perspectives over others [5, 6]. Finally, although theory use is more prevalent in qualitative research, it can also be used in quantitative and mixed methods research as illustrated in the examples we have currated in Table 2. This reinforces the importance of all HPE researchers having some understanding of theory and how to use it. Thus, our second set of considerations relate to considering the interplay between theory and paradigm (summarised in Table 1).

## Consider the interplay between methodology and theory

Methodology refers to the research processes used in a study, encompassing methods of recruitment, data collection and data analysis. An important relationship exists between paradigm and methodology [17], and therefore between methodology and theory. As identified earlier, theory can be used in both qualitative and quantitative HPE research and different methodologies interact differently with theory. For example, traditional grounded theory as a methodology actively rejects pre-existing theory and regards theory development as an endpoint of the research [21]. In contrast, contemporary variants of grounded theory use pre-existing theory to inform the research [22]. Other qualitative methodologies such as hermeneutic phenomenology also use theory to focus the inquiry and explain findings [23]. So, in this context, we encourage ECRs to develop their awareness of the underpinning philosophical foundations of their chosen methodology and its stance on theory, and to engage with examples of published research to identify how others utilising a similar methodology have used theory. Therefore, our third set of considerations (summarised in Table 1) relates to engaging with theory in the context of methodology.

## Consider the fit between theory and unit of analysis

There are two elements relating to the unit of analysis. Firstly, the unit of analysis pertains to the level at which a researcher is intending to examine a research phenomenon. Crotty’s [9] multi-level framework is a useful way to frame the different levels at which a phenomenon can be explored, which is at the level of the: individual or groups (micro-level); organisations/workplaces or medium sized networks (meso-level); or systems or large networks (macro-system). At the micro or individual level, the focus is mainly on the individual and their motivations, learning, performance, and development etc., and theories used at this level include mainly psychological and educational theories. At the micro-level, there can also be a focus on groups or small networks and their interactions, processes, social practices, identity etc., with social psychology, socio-cultural, sociological, social network theories being used. At the meso or organisational level, the focus is on structures and systems within organisations and mid-level networks, and organisational, cultural, socio-cultural, and ecological theories are commonly used at this level. At the macro level, there is a broader focus on systems and large networks, and theories used at this level can include activity theory, systems theory, and complexity theory etc.

Secondly, the unit of analysis can also pertain to whether a researcher is specifically seeking to unravel the impact of power, race, gender, politics, history and culture on phenomena (across micro, meso or macro levels). This would require the use of theories which are critical or emancipatory in nature, such as critical, feminist, intersectional, or postcolonial theory. As discussed earlier, using a critical or emancipatory lens can decolonise and diversify the theoretical knowledge and perspectives represented in the HPE literature [5, 6]. By critically and deeply reflecting on their motivations and values with regards to a research study [17] ECRs can clarify their unit of analysis. As such, our fourth set of considerations (listed in Table 1) relates to identifying the alignment between unit of analysis and theory.

## Consider when theory is used and associated implications

Theory can be used deductively or inductively [2]. When used deductively, theory guides all parts of the process, including conceptualisation, planning and execution of the research [2]. Using theory deductively requires ECRs to outline how the chosen theory has informed the framing of their research problem, the wording of the research aims and questions, and the methods of data collection and analysis. It is important for ECRs to understand that while the deductive approach implies there is a logical and linear way to use theory, initial theoretical understandings are often extended and adjusted as researchers engage in the research process. This includes the processes of collecting and analysing data, applying a theory, critically reflecting on the use of theory, writing up their work [2], and considering the interplay between pre-existing theory and emergent theory [21].

Theory can also be used inductively which involves applying it in the latter stages of data analysis [2]. With this approach, data is first interpreted in an open and exploratory manner using approaches like thematic analysis [24] or framework analysis [25] enabling the identification of preliminary themes. Then a theory-informed analysis is undertaken using a theory that is chosen based on preliminary impressions of the data and engagement with the literature. This inductive approach is also common in secondary analyses where researchers apply theory to interpret research data that has already been collected. With both deductive and inductive approaches, it is important for HPE researchers to show they have engaged critically and comprehensively with theory in justifying their choice of theory (or theories). As such, our fifth set of considerations (outlined in Table 1) relates to considering when theory is used in the research process and associated implications.

## Consider the complexities of how theory is written up

In the HPE research manuscript, theory can be presented in three different ways. In some papers, theory is introduced and described in the Background/Introduction section as a way of framing the research, revisited in the Methods as part of the data collection and analysis, and explained further in the Discussion in terms of the contributions and implications of using theory. This approach is common in both studies where theory is used deductively or inductively, highlighting the complexities of balancing what was done in the research process (which is highly flexible and non-linear) and the reporting of a research study (which demands a logical and linear approach). We discuss this aspect later. In other papers, theory is first presented in the Methods section as a lens for data interpretation and then in the Discussion section as a way of considering the significance of findings and implications. This is common in studies using an inductive approach but may run the risk of reviewers critiquing the transparency and consistency of a paper [26]. Finally, entry-level research mostly only refers to theory in the Discussion section as a way of highlighting the implications of a study, but this approach does not fully leverage the possibilities offered by the use of theory [1–4].

In reporting their work, researchers need to balance between what they have done in the research process (logic-in-use) with how they ‘formulate, articulate, analyse, or evaluate’ what they have done (reconstructed logic) [27]. Logic-in-use is a highly flexible and non-linear process involving juggling between the interrelated elements of ontology, epistemology, methodology, and axiology across the entire research process [9]. In contrast, reconstructed logic involves researchers developing linear and logical narratives about their work in which theory is often presented earlier as part of the framing of a study and separated from methodology and paradigm. Reconstructed logic means that even in studies where theory may have been used inductively and applied at the stage of data collection, a linear narrative can create an impression that the theory was known all along [28]. This highlights the complexities of balancing logic-in-use and reconstructed logic in the write-up phase. As advised earlier, consulting examples of original research articles can help ECRs to discern the different ways in which theory is presented in a research manuscript and how to report theory in a way that is fit for purpose for their research. So, our last set of considerations (outlined in Table 1) relates to considering the complexities of how theory is written up.

## Conclusion

Using theory in HPE research holds significant benefits for the individual researcher, research teams and communities, and the discipline of HPE. Therefore upskilling in theory use is vital for all HPE researchers. We hope the guidance provided here supports new and emerging researchers across the globe to enhance their capacity to discern, enhance, and critique theoretical quality in HPE research.
